# Apelin System in Mammary Gland of Sheep Reared in Semi-Natural Pastures of the Central Apennines

**DOI:** 10.3390/ani8120223

**Published:** 2018-11-28

**Authors:** Francesca Mercati, Margherita Maranesi, Cecilia Dall’Aglio, Linda Petrucci, Rolando Pasquariello, Federico Maria Tardella, Elena De Felice, Paola Scocco

**Affiliations:** 1 Department of Veterinary Medicine, University of Perugia, Via San Costanzo 4, 06126 Perugia, Italy; francesca.mercati@unipg.it (F.M.); margherita.maranesi@unipg.it (M.M.); cecilia.dallaglio@unipg.it (C.D.); linda.petrucci1@studenti.unipg.it (L.P.); 2 Animal Reproduction and Biotechnology Laboratory, College of Veterinary Medicine and Biomedical Sciences, Colorado State University, 1683 Campus delivery, Fort Collins, CO 80523, USA; rolando.pasquariello@gmail.com; 3 School of Biosciences and Veterinary Medicine, University of Camerino, Via Pontoni 5, 62032 Camerino, Italy; dtfederico.tardella@unicam.it (F.M.T.); paola.scocco@unicam.it (P.S.)

**Keywords:** apelin, apelin receptor, mammary gland, *Ovis aries*, Apennine grasslands, drought stress

## Abstract

**Simple Summary:**

Grazing activity is fundamental to natural grassland biodiversity preservation. Increasing summer aridity decreases the grassland pastoral value, negatively affecting animal morpho-functional features and production with detrimental effects on the extensive sheep farming sustainability. Since adipokines represent a link between a subject’s energy availability and tissue metabolism, we investigated the presence and distribution of the system composed of the adipokine apelin and its receptor in the mammary gland of sheep during the period between maximum flowering and maximum dryness of the pasture, providing a group of sheep with food supplementation. This work represents a part of a wider study aimed to buffer the negative effects of increasing summer drought stress on farm income and to maintain the grassland biodiversity. Our findings improve the knowledge of apelin/receptor system function on the sheep mammary gland and this could be a useful tool in the farm management practices.

**Abstract:**

Sheep are the most bred species in the Central Italy Apennine using the natural pastures as a trophic resource and grazing activity is fundamental to maintain the grassland biodiversity: this goal can be reached only ensuring an economical sustainability to the farmers. This study aimed to investigate the apelin/apelin receptor system in ovine mammary gland and to evaluate the differences induced by food supplementation, in order to shed light on this system function. A flock of 15 Comisana x Appenninica adult dry ewes were free to graze from June until pasture maximum flowering (MxF). From this period to pasture maximum dryness (MxD), in addition to grazing, the experimental group (Exp) was supplemented with 600 g/day/head of cereals. Apelin and apelin receptor were assessed by Real-Time PCR and immunohistochemistry on the mammary glands of subjects pertaining to MxF, MxD and Exp groups. They were detected in alveolar and ductal epithelial cells. The pasture maximum flowering group showed significant differences in apelin expression compared with experimental and MxD groups. Apelin receptor expression significantly differed among the three groups. The reduced apelin receptor expression and immunoreactivity levels during parenchyma involution enables us to hypothesize that apelin receptor plays a modulating role in the system control.

## 1. Introduction

The natural and semi-natural pastures in the central Apennines in Italy are used as trophic resources for zootechny, but the increase in summer aridity causes a decrease in the pastoral value of the grasslands; in fact, it is registering an anticipation of the moment of pasture maximum flowering and a shortening of the period between maximum flowering and maximum dryness of pastures. This fact reflects on a lower availability of both forage quality and quantity; the increase in drought stress is therefore detrimental to the sustainability of extensive sheep farms because it affects the morpho-functional features of the animals [1,2,3,4] and greatly reduces milk production [5].

The main income in Italian dairy sheep farms comes from milk (72%), followed by milk-fed or light lamb sales (21%), and subsidies (7%) [6]. So, sheep milk production is an important source of income for livestock breeders and farm economy implementation [5]. Sheep milk, due to its advantageous organoleptic properties, is mainly used for making cheese and dairy products; therefore, it is essential to study the molecular mechanisms and modifications that occur in ovine mammary glands in order to implement milk quality and to improve production strategies.

The mammary gland shows a biological cycle characterized by proliferation, secretion and involution, yet the passage among these three periods/phases is not clear [7]. Indeed, the proliferation of secretory tissue, which occurs during pregnancy, continues during early lactation while involution begins during late lactation, when the alveoli are still producing milk [8]. Mammary development, lactogenesis and mammary involution after the weaning of offspring is regulated by the hypothalamic–pituitary axis in coordination with mammary-released local factors [7]. The mammary gland secretes hormones that can perform local actions including growth hormone, prolactin, parathyroid hormone-related protein, and adipokines. Some adipokines, such as adiponectin, leptin and apelin [9], intervene in mammary growth, function and lactogenic regulation [10,11].

Apelin (APLN) is a novel bioactive peptide belonging to the adipokine family which binds to a specific G protein-coupled receptor named APLNR [12]. Apelin, firstly isolated from the bovine stomach, acts on APLNR localized in the duodenum and colon [13]. Adipose tissue is a potential source of plasma APLN and its secretion by this tissue is regulated by several factors including fasting and refeeding [14]. APLN and APLNR are also widely distributed in the central nervous system and in many peripheral tissues [12]. Therefore, various types of APLN biological activity have been reported, including the regulation of the cardiovascular system, appetite and drinking behavior, gastrointestinal and immune function. This system has been found in mammary gland where APLN mRNA significantly increased during pregnancy and lactation in rats [11]. This system has been investigated in numerous tissues and organs of laboratory animals, human species and, to a lesser extent, bovines. APLN regulates the corpus luteum angiogenesis [15], and it is secreted in colostrum and early milk in bovines [16], so that it is provided to the newborns by nursing and might be involved in gastrointestinal tract development [13].

On the basis of the above-mentioned considerations, this study investigated the presence and expression of APLN and its specific receptor APLNR in the dry sheep mammary gland in order to better understand the actions and functions of APLN/APLNR system. This study is part of multidisciplinary project aimed to buffer the effects of increasing summer drought stress on natural grasslands that represent the main trophic resource for zootechnic purpose in the Central Italy Apennine; so, it was carried out on sheep since this species is the most breed in the study area. Physiological dry period was chosen in order to respect the 3Rs concept of reduction using animals intended for human consumption. Grazing activity is fundamental to maintain the grassland biodiversity but this goal can be reached only ensuring an economical sustainability to the farmers [2,5]. The lack of this condition represents one of the most threats for the pasture abandonment [17], particularly in the 2016 earthquake basin. So, this study was carried out comparing APLN and APLNR expression in sheep grazing on semi-natural pasture during the period between the maximum flowering and maximum dryness of the pasture provided or not with food supplementation, representing an easy and less expensive farmer management action that could be able to buffer the negative effects of drought stress on the animal productivity.

## 2. Materials and Methods

### 2.1. Animal Recruiting, Performances and Tissue Collection

The trial was carried out on 15 Comisana × Appenninica adult female sheep during the dry period, reared in pastures with different drought stress intensities. The ewes were free to graze on a semi-natural pasture of Central Apennine, belonging to the sub-Mediterranean climate, from June to the pasture maximum flowering (MxF). From pasture maximum flowering to maximum dryness (MxD), ten ewes were divided into two homogenous groups as regards age, reproductive performance, body condition score (BCS) and body weight; BCS was assessed also at both the middle and the end of the trial [2]. The MxD group was free to graze on pasture, while the experimental group (Exp) was free to graze on pasture and was also supplemented with 600 g/day/head of barley and corn (1:1), according to the management habits of the farmer (Table S1). Samples were collected from 5 animals for each group: after maximum pasture flowering (early July) (MxF group) and after maximum pasture dryness (early September) (MxD and Exp groups); animals were all dried off from June.

Experimental procedures were approved by the Ministry of Health (No. of approval 95/2018-PR). All animals were intended for human consumption and were slaughtered at the abattoir in accordance with Art. 29 of the Council Regulation (EC) No. 1099/2009 on the protection of animals at the time of killing under law n.333/98 (Council Directive 93/119/EC of 22 December 1993) as specified by Annex C of Section II. Mammary gland samples were collected for carrying out morphological and molecular investigations. For the molecular biology test, samples were thoroughly washed with saline solution, immediately frozen in liquid nitrogen and stored at −80 °C until it was time to measure the transcript expression. For the histological investigations, mammary samples about 1 cm^2^ wide were collected, quickly fixed by immersion in 10% formaldehyde solution in phosphate buffered saline (PBS 0.1 M, pH 7.4) for 36 h and processed until the paraffin wax embedding step.

### 2.2. Morphological Staining and Immunohistochemistry

The fixed samples were dehydrated in graded ethanol, cleared in xylene and embedded in paraffin wax. Serial sections of 5 μm thick were cut and mounted onto poly-l-lysine coated glass slides and air dried at 37 °C. Hematoxylin-Eosin staining was first performed on all specimens to carry out a morphological analysis and to exclude pathologies. Immunohistochemistry was performed as follows [18]: sections were rehydrated, dipped for 10 min in 3% H_2_O_2_ to reduce endogenous peroxidase activity and exposed to microwaves for 15 min at 750 Watts in 0.1 M citrate buffer, pH 6.0, for antigen retrieval. The sections were then blocked with 1:10 normal goat serum (Vector Laboratories, Burlingame, CA, USA) for 30 min and incubated overnight with 1:200 rabbit polyclonal anti-apelin (Novus Biologicals, Littleton, CO, USA) or 1:100 rabbit polyclonal anti apelin receptor (Abcam, Cambridge, UK) antibodies.

On the second day, the sections were incubated for 30 min with 1:200 biotin-conjugated goat anti rabbit secondary antibody (Santa Cruz Biotechnology, Santa Cruz, CA, USA). The bound primary antibodies were visualized using an avidin-biotin system (Vectastain ABC kit; Vector Laboratories, Burlingame, CA, USA) and diaminobenzidine (DAB) as chromogen (Vector Laboratories, Burlingame, CA, USA). The nuclei were counterstained with Mayer’s Hematoxylin. All steps were performed at room temperature and the slides were incubated in a humid chamber. The sections were washed with PBS between all incubation steps, except after normal serum. Sheep abomasum was used as a positive control for both APLN [19] and APLNR [20]. Negative control sections were prepared by omitting the primary antibodies and incubating sections with normal rabbit IgG (Novus Biological, Littleton, CO, USA). All sections were observed under a photomicroscope (Nikon Eclipse E800, Nikon Corp., Tokyo, Japan) connected to a digital camera (Nikon Dxm 1200 digital camera).

### 2.3. RNA Extraction and Real-Time PCR

Total RNA was extracted from the mammary tissue of five sheep for each experimental group as previously described [21]. Five µg of total RNA was reverse transcribed in 20 µL of iSCRIPT cDNA using random hexamer according to the protocol provided by the manufacturer (Bio-Rad Laboratories, Milan, Italy). Genomic DNA contamination was checked by developing a PCR without reverse transcriptase. Serial experiments were carried out to optimize the quantitative reaction, efficiency and CT values. The optimal 25 µL PCR reaction volume contained 12.5 µL of iQ SYBR Green SuperMix (Bio-Rad Laboratories), 1 µL forward and reverse primers (stock concentration of 10 µM) and water to 25 µL. The primers used are listed in Table 1.

All reagents were mixed as a master mix and distributed into a 96-well PCR plate before adding 2 µL of cDNA (10-fold diluted with water). For every PCR run, reaction controls without template and without reverse transcriptase in RT were included as negative controls in order to ensure that RNA was free of genomic DNA contamination. The amplification fidelity of samples was also verified by agarose gel electrophoresis for three animal subjects (Figure S1). The images of gels were acquired by using a Kodak DC290 digital camera. PCR was performed on an iCycler iQ (Bio-Rad Laboratories) with an initial incubation at 95 °C for 1.5 min, followed by 40 cycles at 95 °C for 15 s, 53 °C for 30 s, during which fluorescence data were collected. The threshold cycle (Ct value) was automatically computed for each trace. PCR products were purified and sequenced by Qiaquick PCR Purification Kit according to the manufacturer’s protocols (Quiagen Inc., Milan, Italy). The beta-actin Ct housekeeping gene (ACTB) was determined in order to normalize sample variations in the amount of starting cDNA.

Standard curves were generated by plotting the threshold value (Ct) against the log cDNA standard dilution (1/10 dilution) in nuclease-free water. The slope of these graphs was used to determine the reaction efficiency. Sample mRNA quantification was evaluated using iCycler system software, while mRNA gene expression was quantified using the 2 ^–ΔΔCt^ method [22,23]. The melting curve analysis was carried out, immediately after the PCR end cycle, in order to determine the specificity of each primer set. A melt-curve protocol was performed by repeating 80 heating cycles for 10 s, from 55 °C with 0.5 °C increments, during which fluorescence data were collected.

### 2.4. Statistical Analysis

The experimental unit was the individual animal. The variables tested were mRNA levels of APLN and APLNR. The groups compared (MxF, Exp and MxD) were composed of five experimental units each. We performed a preliminary test of the assumptions for parametric tests by performing Shapiro-Wilk tests of distribution normality (one per group) and Levene’s tests of homogeneity of variance (one for each between-groups comparison). As the data met the assumptions for the parametric tests, we tested the null hypothesis of equality of the means for mRNA levels of APLN and APLNR by performing three 2-tailed *t* tests (MxF vs. Exp, MxF vs. MxD, Exp vs. MxD). Statistical tests were run using R, version 3.4.1, *stats* R package, version 3.4.1 (shapiro. test and *t*. test functions) and *car* R package version 2.1–4 (Levene Test function).

## 3. Results

### 3.1. Animal Performances and Intake

Subjects divided into the MxD and Exp groups at the moment of the maximum pasture flowering showed to have homogeneous characteristics as reproductive performance, body weight and BCS regards (Table 2). Mean values of BCS did not show significant differences between Exp and MxD groups at each observation time: 1.907 ± 0.072 (Standard deviation) vs. 1.913 ± 0.061, *t* = −0.157, df = 8, *p* = 0.880 (8 July 2016); 1.687 ± 0.234 vs. 1.560 ± 0.276, *t* = 0.783, df = 8, *p* = 0.456 (9 August 2016); 1.550 ± 0.194 *vs.* 1.490 ± 0.171, *t* = 0.519, df = 8, *p* = 0.618 (9 September 2016). On the basis of the management kind, it was estimated a mean intake activity able to remove the 15–20% of pasture phytomass during the grazing period, referred to both MxD and Exp groups that were together conducted at the pasture. As Exp group supplementation regards, no refusals remained in the manger.

### 3.2. Morphological and Immunohistochemical Evaluation

**Figure 1:**
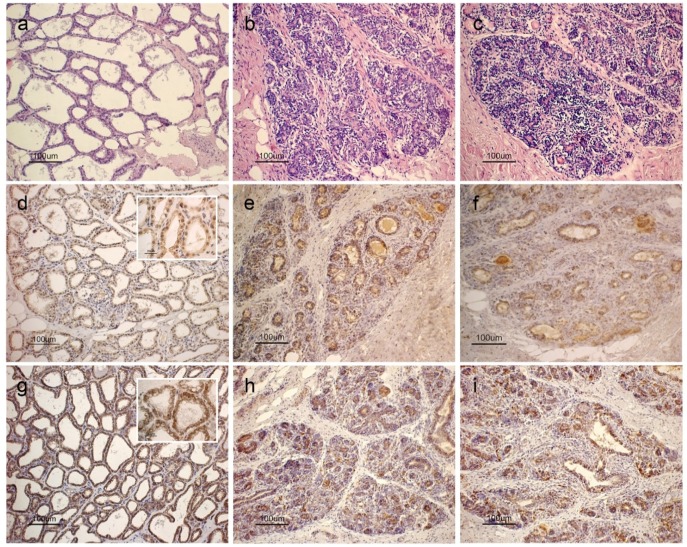
Light micrograph of sheep mammary gland parenchyma. (**a**,**c**) Hematoxylin-eosin staining: (**a**) samples collected at pasture Maximum Flowering (MxF) showed voluminous parenchymal aggregates with broad lumen alveoli; (**b**,**c**) samples collected at the maximum of pasture dryness from both (**b**) Experimental (Exp) and (**c**) Maximum Dryness (MxD) groups showed small alveolar aggregates where alveolar lumen was not visible, and abundant connective tissue. (**d**,**f**) Apelin immunohistochemistry: (**d**) staining is localized to the cytoplasm of epithelial cells as showed by the inset in samples collected at MxF group, nuclei also showed binding patterns; the positivity also persisted in the (**e**) Exp and (**f**) MxD groups even if less widespread. (**g**–**i**) Apelin Receptor immunohistochemistry: (**g**) staining pattern was intense and common to all of the alveolar epithelial cells of MxF group; (**f**) immunostaining appeared weaker in the (**h**) Exp and (**i**) MxD groups.

In the MxF group, the mammary parenchyma mainly showed voluminous alveolar aggregates separated by thin connective septa. Inactive lobules were present; however, some alveoli still showed a broad lumen even if alveolar cells appeared quite low (Figure 1a). In Exp and MxD groups, the parenchyma consisted mainly of small alveolar aggregates, separated by abundant connective tissue with adipose tissue that was especially abundant in the MxD group. The lumen of the alveoli was not visible and the structures clearly appeared inactive with the typical appearance of the late dry period in both Exp and MxD groups (Figure 1b,c).

APLN and APLNR staining was observed in the cytoplasm of alveolar and ductal epithelial cells. APLN showed a clear positivity in MxF mammary gland (Figure 1d); positivity also persisted in the Exp (Figure 1e) and MxD (Figure 1f) groups even if less widespread according to the reduction of mammary parenchyma. A similar staining pattern was observed for APLNR which was intense and common to all of the alveolar epithelial cells of MxF group (Figure 1g); however, immunostaining appeared weaker in the Exp (Figure 1h) and MxD (Figure 1i) groups. No differences were observed between Exp and MxD groups in both the APLN and APLNR staining.

Sheep abomasum, used as positive control, showed binding pattern for both APLN and APLNR (Figure S2a,b), while staining was completely absent in the control sections where the primary antibodies were omitted and in sections incubated with normal rabbit IgG (Figure S2c,d).

### 3.3. Real-Time PCR

**Figure 2:**
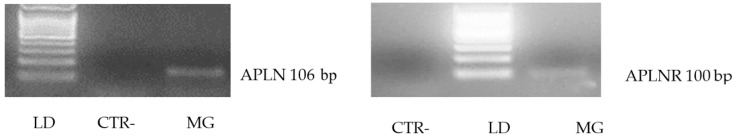
Expression of APLN and APLNR mRNA in sheep mammary gland (MG). Representative agarose gel electrophoresis stained with ethidium bromide to determine whether the expected and obtained PCR products match. For every PCR, a negative control (CTR-) were included, LD = 100 bp DNA ladder.

Gene expression of APLN and APLNR mRNA in sheep mammary gland were evidenced in Figure 2.

In addition, the Real-Time PCR assay highlighted the presence of the transcripts for both APLN and APLNR (Table 3) in the mammary tissue of all groups. As APLN concerns, the maximal mRNA abundance was found in MxF group that showed a significant difference compared to the MxD and Exp groups; while no differences emerged between MxD and Exp groups. Moreover, APLNR mRNA was most abundant in the MxF group, but significant differences were observed between all the three groups.

## 4. Discussion

Farm management kind, able to remove only the 15–20% of pasture phytomass, seems to be not very effective for both pasture suitable exploitation and biodiversity maintaining; in addition, the observed mean BCS values of both MxD and Exp groups showed to be lesser than the threshold value considered sufficient to maintain animal wellbeing only in not for breading/milking subjects [24]. Food supplementation did not affect the animal body status, as suggested by the absence of significant differences in the mean values of BCS during the experimental trial. BCS was followed during the trial because it is considered a more sensitive indicator of the animal nutritional status than body weight, which is affected by the gastrointestinal content weight [25].

Food supplementation produced differences on the mammary gland morpho-functional features of the three animal groups. The mammary gland has a compound tubulo-alveolar structure [26]; in the active gland, the parenchyma is divided into lobules made of groups of adenomeres, separated by thin connective septa containing vessels and nerves. The parenchyma regresses in the period between two lactation cycles: the alveoli lose the lumen or disappear while the excretory ducts persist longer; the connective septa thicken and accumulate adipose tissue. The adenomere wall is composed by a simple prismatic epithelium whose thickness varies according to the functional stage of the gland [7]. Secretory tissue grows during late gestation and early lactation and the modifications the epithelial cells undergo are essential for milk production. It is known that the highest percentage of epithelial tissue and proliferating epithelia cells is around d115 of gestation; this period is also associated with increase in epithelial cell size and a general appearance of secretory activity [27]. During lactation, the epithelial cells are low columnar in shape and have secretory vesicles, yet they become attenuated cuboidal in the dry period. The nonlactating period between drying off and parturition is also an important period of renewal in dairy animals, with the first thirty days of the dry period considered to represent a phase of active involution, and the subsequent thirty days a period of cellular renewal [28]. The observed morphological pictures, indicate that the mammary gland is in an early period of involution in the MxF group, while both in MxD and Exp ones, mammary gland shows the typical inactive aspect of the dry period.

Findings also demonstrated the presence in the sheep mammary gland of APLN and APLNR; in particular, there is the major expression of APLN in ovine mammary glands during the pasture maximum flowering (MxF group), when the mammary gland is by short time in the dry period; while the APLN expression decreases during the pasture maximum pasture dryness (MxD and Exp groups) when the parenchyma shows the most involution. In the rodent mammary gland APLN increases during pregnancy and lactation, reaching its maximum level at parturition and decreases gradually during lactation [11,29]. In our study, the APLN expression appeared to decrease according to the degree of postpartum breast involution. In ruminants, mammary epithelial cells do not regress to the same extent as in rats after weaning [11,30] moreover, the mammary gland maintains some synthetic and secretory activity during the non-lactating phase [31,32].

Immunohistochemical findings showed unmodified intensity in APLN staining, even if the binding patterns were less widespread according to the decrease in adenomere development. The apparent incongruity between immunohistochemical and molecular data may be due to a different degree of sensitivity of the two techniques adopted and explained with the hypothesis that APLN is partly secreted in exocrine mode into the ductal system in the MxF group, as suggested by other authors [11,16,33]. The constant immunopositivity to APLN in mammary gland may be attributed to the APLN secreted through paracrine and autocrine mechanisms as already observed for other tissues [13,33,34] or species [35]. The role as paracrine and autocrine hormone for APLN is strictly related to local interaction with its specific receptor APLNR while the molecule is not delivered to other tissues by circulation [33,35]. Kleinz et al. [36] reported the localization of apelin immunoreactivity accumulated in structures closely associated to the nuclear surface; the immunopositivity we observed at nuclear level could be associated to this described case.

APLNR binding patterns were evidenced at alveolar and ductal cell cytoplasm level; APLNR is a transmembrane receptor belonging to the G protein-coupled family receptors. These receptors can be phosphorylated and internalized inside the cell cytoplasm as already described [37,38]. The APLNR in ovine mammary glands is differently expressed in the MxF, Exp and MxD groups, in which it decreases respectively. Immunohistochemistry shows different staining patterns for this molecule that is strongly reactive in the MxF group and slightly positive in both the MxD and Exp groups. However, the changes of the APLNR expression observed between the three groups under study suggest that this molecule may play the main role in the APLN/APLNR system in ovine mammary glands, by acting as system modulator.

In this perspective, supplementation may have had a positive effect by maintaining the mammary gland active for longer after the end of lactating period. This hypothesis is partly supported by the fact that, after using the same experimental protocol on lactating ewes, a high decrease in milk production was observed in MxD group, while the Exp group maintained significantly higher milk yields for a certain time [5]. It may be assumed that the major milk production induced by supplementation maybe due to higher levels of APLNR expression, as observed in the dry Exp group. Indeed, supplementation induced higher APLNR expression may maintain a higher milk production in lactating subjects, while it may delay mammary gland involution in dry subjects, as actually observed.

## 5. Conclusions

The knowledge of each mammary gland regulatory system could be a useful tool to support farm productivity; the food supplementation seems to improve the sheep mammary gland functionality and, at the same time, it represents a less expensive and easy management action for the farmer, that usually use a mix of cereals to improve the flock feeding. To the best of our knowledge this is the first study on the expression of APLN and APLNR in the mammary glands of ewes during the dry period. Even if the investigation involved a reduced number of subjects, the evaluation of APLN and APLNR levels and the immunolocalization in the alveolar mammary epithelium strongly suggests that the APLN/APLNR system may be involved in the functioning of ovine mammary glands. Moreover, APLNR may play an important role as system modulating molecule, therefore regulating mammary gland activity. Research is in progress to confirm obtained findings in a greater number of animals and in different sheep breeds.

## Figures and Tables

**Table 1:** Primers for apelin, apelin receptor and beta-actin (used as internal standard) for Real Time-PCR quantification.

Gene		Primers	bp
APLN	F	CTTCTGACGGGAAGGAGATG	106
	R	CGGAACTTCCTCCGACCT	
APLNR	F	TTGTGGGTCTGGAGGGTAAG	100
	R	GCTGGGAGCATTTCAGAGAC	
ACTB	F	CCTTAGCAACCATGCTGTGA	130
	R	AAGCTGGTGCAGGTAGAGGA	

APLN: apelin; APLNR: apelin receptor; ACTB: beta-actin; bp: base pair.

**Table 2:** Animal performances.

Items	MxD	Exp
Animal Age (years)	3	3
Number of pregnancies for each subjects	2	2
Number of lamb/pregnancy for each subjects	1	1
Mean Body weight (Kg)	50.12	49.88
Mean Body Condition Score	1.913	1.907

**Table 3:** Mean mRNA levels and standard deviation values (SD) of apelin and its receptor in sheep mammary gland.

	APLN		APLNR	
Groups	Mean	SD	Mean	SD
MxF	6.201	0.454	0.839	0.254
Exp	1.613	0.112	0.229	0.030
MxD	1.620	0.178	0.184	0.043
*P_MxF vs. Exp_	1.98 × 10^−8^		0.000672	
*P_MxF vs. MxD_	2.79 × 10^−8^		0.000438	
*P_MxD vs. Exp_	0.9508		0.07745	

APLN: apelin; APLNR: apelin receptor; MxF: maximum flowering group; Exp: experimental group; MxD: maximum dryness group; * Significance values (*P*) between groups comparisons, carried out with the *t* test.

## Supplementary Materials

The following are available online at http://www.mdpi.com/2076-2615/8/12/223/s1, **Figure S1:** Expression of apelin (APLN) (A) and apelin receptor (APLNR) (B1 and B2) mRNA in sheep mammary gland (MG) belonging to three animals (MG1, MG2, and MG3) in the different experimental conditions (MxF = maximum of pasture flowering; Exp = experimental group; MxD = control group); **Figure S2:** (a) APLN and (b) APLNR immunohistochemistry in the abomasum of the sheep; **Table S1:** Composition of the feed supplementation (%).
