# Supplementary material for: Apelin System in Mammary Gland of Sheep Reared in Semi-Natural Pastures of the Central Apennines
Source: Animals (Basel). 2018 Nov 28;8(12):223. doi: 10.3390/ani8120223 (PMC6315652; doi:10.3390/ani8120223)
Supplement: Supplementary file 1 [file animals-08-00223-s001.pdf]

Apelin system in mammary gland of sheep reared in semi-natural pastures of the Central Apennines

F. Mercati, M. Maranesi, C. Dall'Aglio, L. Petrucci, R. Pasquariello, F.M. Tardella, E. De Felice, P. Scocco

Material and methods

Animal recruiting, performances and tissue collection

The trial was carried out on 15 Comisana x Appenninica adult female sheep during the dry period, reared in pastures with different drought stress intensities. The ewes were free to graze on a semi-natural pasture from June to the pasture maximum flowering. From pasture maximum flowering to maximum dryness, ten ewes were divided into two homogenous groups as regards age, reproductive performance, body condition score (**BCS**) and body weight; BCS was assessed also at both the middle and the end of the trial [1]. The maximum dryness group (**MxD**) was free to graze on pasture while the experimental group (**Exp**) was free to graze on pasture and was also supplemented with 600g/day/head of barley and corn (1:1) (Supplementary Table S1); this type of supplementation was chosen according to the farmer, because it is usually adopted when there are some problems in the animal feed, so it doesn't represent an additional management weigh. Samples were collected after maximum pasture flowering (early July) (**MxF** group) and after maximum pasture dryness (early September) (MxD and Exp groups).

All animals were intended for human consumption and were slaughtered at the abattoir in accordance with Art. 29 of the Council Regulation (EC) No. 1099/2009 on the protection of animals at the time of killing under law n.333/98 (Council Directive 93/ 119/EC of 22 December 1993) as specified by Annex C of Section II. Mammary gland samples were collected for carrying out morphological and molecular investigations. For the molecular biology test, samples were thoroughly washed with saline solution, immediately frozen in liquid nitrogen and stored at −80°C until it was time to measure the transcript expression. For the histological investigations, mammary samples about 1 cm^2 wide were collected in the middle portion of the mammary gland one centimeter from the dorsal boundary, quickly fixed by immersion in 10% formaldehyde solution in phosphate buffered saline (PBS 0.1 M, pH 7.4) for 36 h and processed until the paraffin wax embedding step.

Morphological staining and Immunohistochemistry

Haematoxylin-Eosin staining was performed on all specimens to carry out a morphological analysis and to exclude pathologies.

Immunohistochemistry was performed with the following primary antibodies: rabbit polyclonal anti-apelin (Novus Biologicals, Littleton, CO, USA) and rabbit polyclonal anti apelin receptor (Abcam, Cambridge, UK).

The development of the immunoreaction was performed with biotin-conjugated goat anti rabbit secondary antibody (Santa Cruz Biotechnology, Santa Cruz, CA, USA); avidin-biotin system (Vectastain ABC kit; Vector Laboratories, Burlingame, CA, USA) and diaminobenzidine (DAB) as chromogen (Vector Laboratories, Burlingame, CA, USA).

Sheep abomasum was used as a positive control for both APLN [2] and APLNR [3]. Negative control sections were incubated with normal rabbit IgG (Novus biological, Littleton, CO, USA).

RT-PCR and Gel electrophoresis

Total RNA was extracted from the mammary tissue of three sheep for each experimental group (MxF, Exp, and MxD). Five µg of total RNA was reverse transcribed in 20 µl of iSCRIPT cDNA (Bio-Rad Laboratories, Milan, Italy) using random hexamer protocol.
Genomic DNA contamination was checked by developing a PCR without reverse transcriptase. The multiplex PCR amplification was performed using 1.0 µL of complementary DNA as a template for APLN and APLNR primers (Table 1 main text).
Cycling conditions consisted of an initial denaturizing cycle at 94°C for 75 s, followed by 30 cycles for each gene at 94°C for 15 s, 60°C for 30 s, 72°C for 45 s, and a final extension step at 72°C for 10 min. Within each experiment, the complete set of samples was processed in a single PCR for APLN (Figure S1 A), and in two different PCR for APLNR (Figure S1 B1 and B2). The amplified PCR-generated products (18 µL of 25 µL total reaction volume) were analyzed by electrophoresis on 2% agarose gel using ethidium bromide staining.

Table S1. Composition of the feed supplementation (%)

Dry matter	88.5
Crude protein	10
Lysine	0.3
Methionine	0.16
Tryptophan	0.09
Fat	3.0
Crude fibre	3.7
Ash	1.8
Ca	0.03
P	0.31
Starch	57.5
Free sugars	2.1
NDF (% DM)	12.5
Lignin	1.3
UFL (energy)	1.05

A

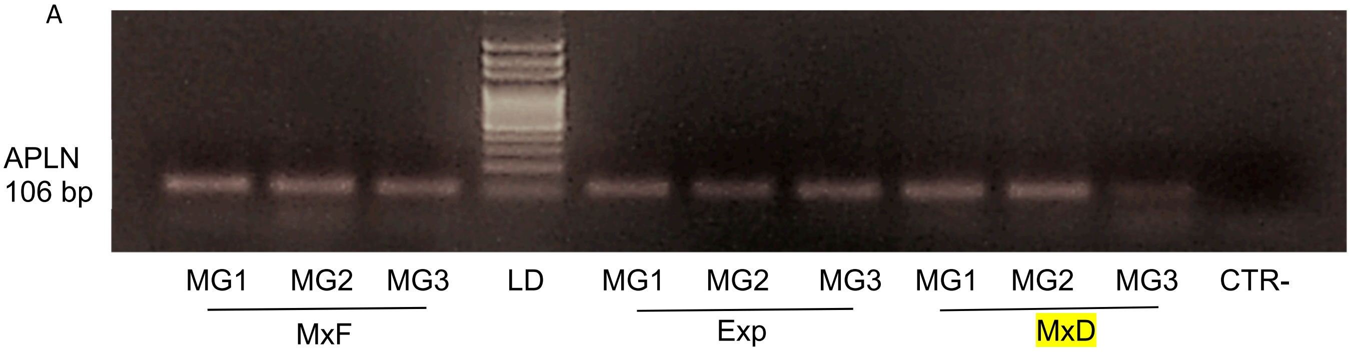

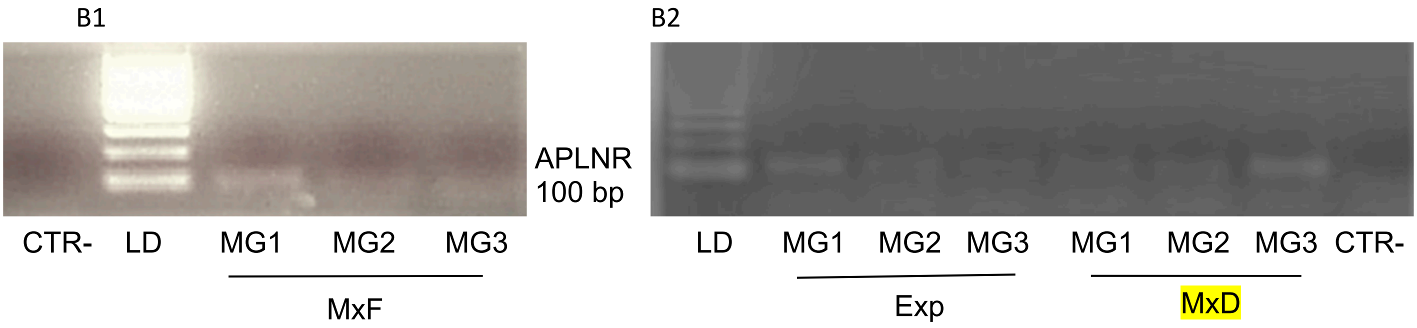

Figure S1 Expression of apelin (APLN) (**A**) and apelin receptor (APLNR) (**B1** and **B2**) mRNA in sheep mammary gland (MG) belonging to three animals (MG1, MG2, and MG3) in the different experimental conditions (MxF= maximum of pasture flowering; Exp= experimental group; MxD= maximum of pasture dryness). Representative agarose gel electrophoresis stained with ethidium bromide to verify matching between expected and obtained PCR products. Within each experiment, the complete set of samples was processed in a single PCR for APLN (**A**), and in two different PCR for APLNR (**B1** and **B2**). For every PCR, a negative control was included (CTR-), LD = 100 bp DNA ladder.

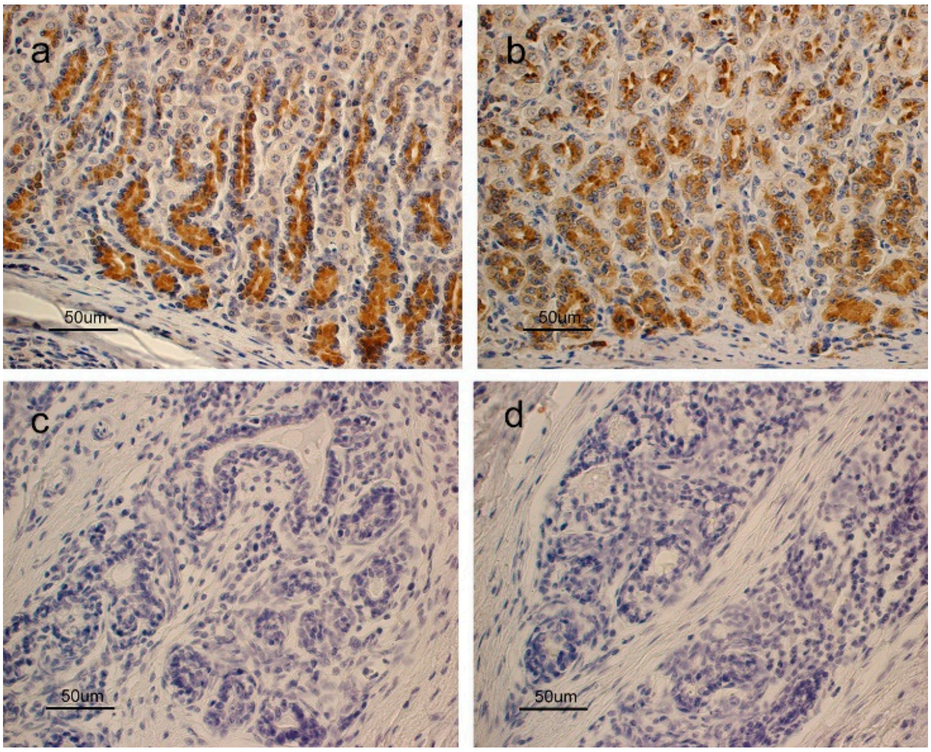

Figure S2 (**a**) APLN and (**b**) APLNR immunodetection in the abomasum of the sheep. Staining is mainly localized in the basal region of the gastric glands. Abomasum was used as positive control for the immunohistochemical assays. Mammary negative control for (**c**) APLN and (**d**) APLNR obtained with normal rabbit IgG incubation instead of primary antibody. Epithelial cells of the alveoli and ducts are negative. In all sections, the nuclei were counterstained with Mayer's Haematoxylin.

References

1. Scocco, P.; Piermarteri, K.; Malfatti, A.; Tardella, F.M.; Catorci, A. Increase of drought stress negatively affects the sustainability of extensive sheep farming in sub-Mediterranean climate. *J. of Arid Environ.* **2016**, *128*, 50–58. https://doi.org/10.1016/j.jaridenv.2016.01.006
2. Susaki, E.; Wang, G.; Cao, G.: Wang, H.Q.; Englander, E.W.; Greeley, G.H. Jr. Apelin cells in the rat stomach. *Regul. Pep*., **2005**, *129*, 37-41. https://doi.org/10.1016/j.regpep.2005.01.013
3. Lambrecht, N.W.; Yakubov, I.; Zer, C.;Sachs, G. Transcriptomes of purifiedgastric ECL and parietal cells: identification of a novel pathway regulating acid secretion. *Physiol. Genomics*, **2006**, *25*, 153-65. https://doi.org/10.1152/physiolgenomics.00271.2005
